# Prenatal Stress and Peripubertal Stimulation of the Endocannabinoid System Differentially Regulate Emotional Responses and Brain Metabolism in Mice

**DOI:** 10.1371/journal.pone.0041821

**Published:** 2012-07-25

**Authors:** Simone Macrì, Chiara Ceci, Rossella Canese, Giovanni Laviola

**Affiliations:** Department of Cell Biology and Neurosciences, Istituto Superiore di Sanità, Roma, Italy; Radboud University, Netherlands

## Abstract

The central endocannabinoid system (ECS) and the hypothalamic-pituitary-adrenal-axis mediate individual responses to emotionally salient stimuli. Their altered developmental adjustment may relate to the emergence of emotional disturbances. Although environmental influences regulate the individual phenotype throughout the entire lifespan, their effects may result particularly persistent during plastic developmental stages (e.g. prenatal life and adolescence). Here, we investigated whether prenatal stress – in the form of gestational exposure to corticosterone supplemented in the maternal drinking water (100 mg/l) during the last week of pregnancy – combined with a pharmacological stimulation of the ECS during adolescence (daily fatty acid amide hydrolase URB597 i.p. administration - 0.4 mg/kg - between postnatal days 29–38), influenced adult mouse emotional behaviour and brain metabolism measured through in vivo quantitative magnetic resonance spectroscopy. Compared to control mice, URB597-treated subjects showed, in the short-term, reduced locomotion and, in the long term, reduced motivation to execute operant responses to obtain palatable rewards paralleled by reduced levels of inositol and taurine in the prefrontal cortex. Adult mice exposed to prenatal corticosterone showed increased behavioural anxiety and reduced locomotion in the elevated zero maze, and altered brain metabolism (increased glutamate and reduced taurine in the hippocampus; reduced inositol and N-Acetyl-Aspartate in the hypothalamus). Present data further corroborate the view that prenatal stress and pharmacological ECS stimulation during adolescence persistently regulate emotional responses in adulthood. Yet, whilst we hypothesized these factors to be interactive in nature, we observed that the consequences of prenatal corticosterone administration were independent from those of ECS drug-induced stimulation during adolescence.

## Introduction

Individual emotional regulations depend on a continuous cross-talk between biological predispositions and environmental challenges [1], [2]. Epidemiological evidence, and clinical and preclinical studies demonstrate that environmental stimulation regulates individual emotional reactivity throughout the entire lifespan [3], [4]. The regulatory role exerted by the environment seems to occur primarily during those developmental stages characterized by an elevated degree of phenotypic plasticity, defined as the “phenotypic modifications that may be expressed by a given organism under contrasting conditions” [5], [6]. A large body of experimental evidence indicates that environmental stress occurring early in life is capable of persistently adjusting emotional regulations between infancy through adulthood [7]–[9]. For example, Maccari and Morley-Fletcher reported that severe stressors occurring during gestation may persistently up-regulate behavioural and endocrine indices of stress, fear and anxiety in humans and in rodents [10] (see also [11]). Environmentally mediated variations of individual phenotype have also been proposed to potentially relate to altered function of reward pathways and, in turn, favour the onset of drug-related phenotypic abnormalities in adult rats [12]–[16]. Just as a series of studies highlight the elevated sensitivity to context characterizing the very early stages of life [1], [17], so also numerous observations indicate that other developmental phases are characterised by an elevated degree of plasticity (e.g. [3], [18]). Adolescence constitutes one of these stages whereby it is characterised by massive restructuring at the level of neuronal connectivity [19]–[21] and is particularly responsive to environmental influences [22], [23]. Thus, together with influencing individual long-term regulations early in development, external challenges may persistently adjust individual stress and fear reactivity also if occurring during adolescence [3].

Along with the phenotypic description of stress-induced alterations at the neuronal, endocrine and behavioural level, several studies attempted to elucidate their biological determinants. A large proportion of these studies focussed on the hypothalamic-pituitary-adrenocortical (HPA) axis (and its effectors) as a principal mediator of the environmental influences on individual plastic regulations [10], [24]. Such focus related to the fact that the HPA axis is instantaneously activated in response to the onset of a stressor [25]. These studies revealed that the aforementioned short-term responses translate into persistent modifications in the reactivity of the axis itself (e.g. glucocorticoid receptor expression, [24], adrenal sensitivity to ACTH stimulation [26]).

Beside the HPA axis, other biological systems play a fundamental role in the regulation of emotions. Among these, the endocannabinoid system (ECS) has emerged as a fundamental regulator of emotional reactivity to stressful events [27], [28]. The ECS is composed of cannabinoid receptors (CB1 and CB2), their endogenous ligands (AEA and 2-AG) and the enzymes that orchestrate their synthesis and degradation (e.g. the fatty acid amide hydrolase, FAAH). Specifically, several authors reported that the ECS responds to both acute and chronic stressors in rats [28], [29]. Thus, Rademacher and colleagues [30] observed that adult mice exposed to repeated 30-min restraint stress sessions showed acute and chronic fluctuations in AEA and 2-AG concentrations in selected brain areas. Hill and McEwen [27] recently described the basic mechanisms linking ECS and HPA activation in response to stressful challenges (see also [31]). Beside the observation of short- and long-term ECS adaptations in response to stressful stimuli in adulthood, experimental data revealed that the ECS is rapidly activated in response to stressful stimuli in infancy. For example, we recently observed that the provision of social stimulation (an unfamiliar age- and weight-matched conspecific) to adolescent rats was sufficient to induce a short-term secretion of anandamide within the striatal brain area [32]. Beside the observation of short-term activation in response to external stressors, several studies demonstrate that developmental exposure to environmental challenges persistently regulates the ECS. For example, neonatal stress has been shown to exert long-term influences at the level of ECS ligands, enzymes, and receptors [33], [34]. Thus, a large body of experimental evidence suggests that the HPA axis and the ECS may be directly involved in long-term developmental regulations of emotional reactivity and that they may orchestrate both physiological and pathological adaptations [35].

Here we attempted to investigate the role of prenatal stress and of an exogenous modulation of the ECS activity during adolescence in the long-term regulation of emotional responses in rodents. Specifically, we hypothesised that prenatal stress and the pharmacological stimulation of the ECS during adolescence may exert additive effects in persistently affecting emotional reactivity throughout development. To address this hypothesis we supplemented mouse dams with corticosterone in their drinking water during the last third of pregnancy – through a well-validated methodology [36], [37] – and then exposed their adolescent offspring to repeated i.p. injections of URB597, an indirect AEA agonist acting through the inhibition of FAAH. Whilst the corticosterone supplementation served to mimic the physiological events characterizing a chronic stressor applied to the pregnant mothers [36]–[38], the URB597 administration during adolescence served to artificially maintain elevated AEA levels that are naturally secreted on demand [39]. We decided to administer corticosterone during the last third of pregnancy based on the following grounding: (1) this selection allowed direct comparison with a large body of studies investigating the effects of different forms of prenatal stress during this specific time window [10], [40]–[42]; (2) since the expression of mineralocorticoid receptors in mice is particularly elevated around this developmental stage [43], a stressor applied during this period may exert profound long-lasting consequences on emotional regulations. We then addressed the following parameters in developing male mice: general locomotion in response to acute and semi-chronic administration of URB597 during adolescence (postnatal days, P 29 to 38), behavioural anxiety through the elevated 0-maze test (P80), general locomotion through a classical open-field test (P100), anhedonia through a fully automated progressive-ratio test (P140–160), and indices of brain metabolism through *in vivo* non-invasive magnetic resonance imaging-guided spectroscopy (MRI/MRS) in behaviourally-relevant brain areas.

## Materials and Methods

### Animals

**Figure 1:**
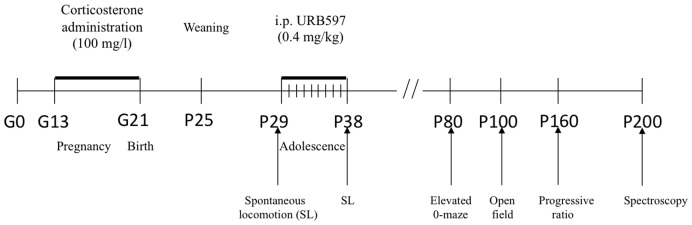
Timeline of the experiment. It shows the age of animals during gestation (G) or during postnatal life (P) at which experimental procedures (treatment and/or testing) have been conducted (see Materials and Methods for details).

Twenty pregnant outbred CD-1 mice (purchased from Charles River®, Italy) were housed in standard polycarbonate cages (33.0×13.0×14.0 cm) with sawdust bedding and *ad libitum* water and rodent pellets (Enriched standard diet purchased from Mucedola, Settimo Milanese, Italy). They were maintained on a reversed 12∶12 h light:dark cycle (lights on at 1900 h) with temperature at 21±1°C and relative humidity of 60±10%. Dams were inspected daily at 0930 h for delivery and day of birth was designated as postnatal day 1 (P1). Between delivery and weaning (P25) all subjects were kept under standard facility rearing conditions (cage cleaning once a week). Litters were not culled until weaning and dams that delivered less than six pups or litters in which male to female ratio was heavily skewed in one or the other direction (more than 75% of same sex pups) were excluded from the experiment. At weaning, each dam contributed 3–4 male offspring, which were allocated in cages of two unrelated subjects (one AFR and one PNC). Out of these four subjects, two were allocated to the VEH group and two were allocated to the URB597 group. All experimental procedures were performed according to European Communities guidelines (EC Council Directive 86/609), Italian legislation on animal experimentation (Decreto L.vo 116/92) and NIH guide for the care and use of laboratory animals. The study has been approved by the Service for Biotechnology and Animal Welfare of the Istituto Superiore di Sanità and authorized by the Italian Ministry of Health (Decree Nr. 217/2010-B). A general timeline of the study is reported in Figure 1.

### Prenatal Corticosteone Administration

Pregnant dams were randomly assigned to one of the following prenatal conditions: (1) Animal Facility Rearing (AFR, N  = 10): apart from weekly cage cleaning dams where kept in the animal housing room without being handled or manipulated; (2) prenatal corticosterone administration (PNC, N  = 10) between gestational days 14–21 mice had free access to a solution of 100 µg/ml of corticosterone 21-hemisuccinate [44]. During this period, dams’ fluid intake was monitored every second day. We decided to measure fluid intake every second day, instead of every day, in order to keep the disturbance of the cage to a bare minimum. Thus, fluid intake was measured when drinking bottles were refilled. Other than solution in the drinking bottle (water or corticosterone) environmental conditions were identical among groups. Only male offspring were used in the study.

### Drug Administration during Adolescence

URB597 (cyclohexyl-carbamic acid 3′-carbamoyl-biphenyl-3-yl ester) (Alexis, San Diego, CA, USA) was dissolved in Tween-80 (5%), and 0.9% saline (95%) and administered i.p. at a volume of 0.1 ml/kg body weight. All animals were injected once daily for 10 consecutive days during adolescence (P29–38) with vehicle (VEH) or URB597 (0.4 mg/kg).

### Locomotor Activity: Apparatus and Schedule

Spontaneous locomotor activity in the home-cages was monitored continuously for 5 hours the first and last day of URB597 administration, starting 35 minutes after the URB597 injection. Daily spontaneous activity was monitored by means of an automatic device using small passive infrared sensors positioned on the top of each cage (ACTIVISCOPE system, NewBehaviour Inc., Zurich, Switzerland). The sensors (20 Hz) detected any movement of mice with a frequency of 20 events per second. Data were recorded by an IBM computer with dedicated software. No movements were detected by the sensors when mice were sleeping, inactive, or performed moderate self-grooming. Scores were obtained during 1-h intervals and expressed as counts per minute (cpm). The position of cages in the rack was such that mice of each group were equally distributed in rows and columns. The access of the authorized personnel to the animal room was not restricted and followed the routine schedule. The floor of the apparatus was cleaned after each mouse was tested, and the test was performed under dim illumination.

### Elevated 0-maze (E0M) Test

To evaluate the exploration of an environment imposing on the animal an approach-avoidance conflict, mice were tested on the elevated 0-maze, a paradigm originally described by [45]. We recorded the following parameters: spatiotemporal measures comprised the frequencies of sector entries (sector entries were defined as the animal entering the respective sector with all four paws) and the time spent in the open or closed parts of the maze in 5 min. Furthermore the latency to the first open sector entry was scored. We used the number of entries into and the time spent in the open sectors as an inverse index of anxiety [45]. The sessions started placing the animal in one of the closed sectors. The floor of the apparatus was cleaned with a 10% ethanol 90% water solution after each session. Testing occurred under dim lights. The testing sessions were videotaped and the behavioural profile was subsequently scored by a trained observer, blind to treatments, using a computer and a dedicated software (THE OBSERVER 2.0, Noldus Information Technology, Wageningen, The Netherlands).

### Open Field Activity in Adulthood

Approximately two months after the last URB597 injection, general locomotion was measured during a 30-min open-field test session. The testing apparatus consisted of a squared arena (40×40 cm) with a 50 cm-high wall made of black plastic. The session, performed under indirect dim light, started placing the animal in one corner of the arena. The floor of the apparatus was cleaned with a 10% ethanol 90% water solution after each session. Behavioural analysis was performed by a trained observer, using a computer and dedicated software (the Ethovision 3.0, Wageningen, The Netherlands). This allowed a detailed analysis of the following parameters: latency to access, frequency of entries, and time spent in the centre of the arena (20×20 cm); absolute levels of general locomotion.

### Progressive Ratio Operant Procedure

At age 5–6 months, mice were trained and tested in the progressive ratio (PR) schedule for reinforcement. We obtained three measures of reward motivation, namely, the total number of nose-pokes responses, total number of reinforcements obtained and the final ratio achieved/breakpoint. The apparatus, general schedule and food restriction procedures have been described elsewhere [46]. In order to increase individual motivation to perform the operant responses, the general procedure entailed a 20-hr food restriction regime, beginning one day before the first training session. The inclusion of repeated training sessions (fixed ratio, FR1 and FR3) allowed mice to habituate to the food restriction regime before the beginning of the progressive ratio schedule. When a mouse performed the necessary number of nose pokes, a single pellet dropped from a reservoir into the reward magazine and the magazine-light lit for 5s while the hole-light turned off. During this 5s interval the hole was inactive (nose pokes performed in that time interval were not counted). Each chamber was independently connected to a personal computer. A dedicated software controlled the sessions and recorded the behavioural data.

Following the acquisition of a stable level of nose poking on the FR3 schedule, the animals were transferred to the PR schedule, which requires the subjects to emit an increasing number of responses in order to obtain each reward. The ratios used in this experiment increased as follows: 3, 3, 6, 6, 10, 15, 21, 28, 36, 45, 55, 66, 78, 91, 105, 120, 136, 153, 171, 190, 210, 231, 253, 276, 300, 325, 351, 378, 406, and 435. The highest ratio achieved by each animal during a session represented its breakpoint value. The testing session began by placing the mouse into the chamber and terminated when subjects failed to reach the next nose-poke criterion within 8 min. Values for these measures correlated highly with each other and the final ratio achieved/breakpoint is presented.

### Magnetic Resonance Imaging and Spectroscopy

**Figure 2:**
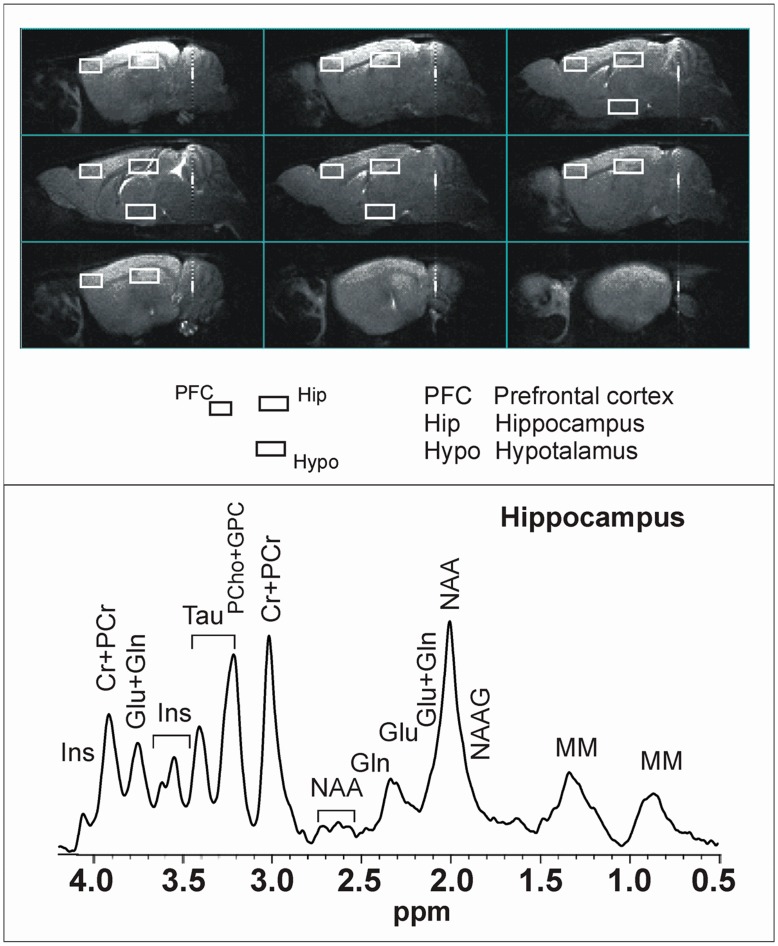
MRI/MRS analysis for brain metabolic changes. **Upper panel:** example of in vivo sagittal T2-weighted spin-echo images (TR/TE = 3000/70 ms, slice thickness 0.8 mm, NS = 2, FOV = 20×20 mm2, matrix 128×128). Voxels localised on prefrontal cortex (PFC), hippocampus (Hip) and hypothalamus (Hypo) are indicated by the white rectangles. **Lower panel:** examples of in vivo 1H spectra, acquired from the Hip (PRESS, TR/TE = 4000/23 ms, NS = 256). Metabolite assignments: Ins, inositol; Cr, creatine; PCr, phospho-creatine; Gln, glutamine; Glu, glutamate; Tau, taurine; PCho, phospho-choline; GPC, glicero-phospho-choline; NAA, N-acetyl-aspartate; NAAG, N-acetyl-aspartyl-glutamate; MM, macromolecules.

At adulthood (>P160), the animals underwent MRI/MRS analyses, with the aim to measure any long-term modifications in biochemical parameters in relevant brain areas. All MRI and MRS experiments were conducted on a 4.7 T Varian Inova animal system (Varian/Agilent Inc. Palo Alto, CA, USA), equipped with actively shielded gradient system (max 200 mT/m, 12 cm bore size). A 6-cm diameter volume coil was used for transmission in combination with an electronically decoupled receive-only surface coil (Rapid Biomedical, Rimpar, Germany). The shape of this receiver coil (1.5 cm long, 1.5 cm wide and 0.6 cm high) was designed to optimally fit the dorsal surface of the mice’ head, centered over forebrain regions. Animals were anaesthetised with isofluran 1.5–2.5% in O_2_ 1 l/min (Isoflo, Esteve Veterinaria, Milan, Italy) and maintained to the animal body temperature at 37.0±0.1°C by an integrated heating system. Gradient echo scout images were detected for accurate positioning of the head of the animal inside the magnet and spin echo sagittal anatomical images (TR/TE = 3000/60 ms, 13 consecutive slices of 0.8 mm thickness, FOV = 20×20 mm^2^, matrix of 128×128, 2 averages) were acquired for positioning the voxel for the MRS study. Single voxel localised ^1^H MR spectra (PRESS, TR/TE = 4000/23 ms, ns = 256 or 512) were collected from relevant brain areas: prefrontal cortex (PFC, 6.8 µl), hippocampus (Hip, 11.7 µl) and hypothalamus (Hypo, 8.8 µl) as shown in Fig. 2. Quantitative MRS protocol, including water T2 measurements, was applied [47]. Localised shimming was performed up to water linewidths smaller or equal to <12 Hz. Long TR and short TE parameters were selected in our pulse sequences, in order to minimize the error due to any potential carry-over influence of adolescent drug treatment on the relaxation times, and therefore to be able to attribute any change in signal intensities to actual changes of metabolite levels. Nevertheless, T2 measurements were performed on water signal in order to identify any change due to treatment (a set of water localised spectra was acquired from the same voxel with the same parameters but TE ranging from 23 to 300 ms, 15 values). The water signal was suppressed by using the VAPOR pre-sequence composed by seven CHESS pulses with optimized flip angles and timing in order to have a reduced sensitivity to B1 variation and thus it is highly efficient also for surface coil; 256 averages were sufficient to acquire metabolite spectra from PFC, STR and Hip with a S/N (referred to the highest signal) higher than 4; hypothalamic spectra required 512 averages due to the distance of this voxel from the coil. Unsuppressed water signal was acquired from the same voxel with the same parameters except for a reduced number of transients (4 instead of 256) and was used for metabolite quantification (assuming 79.9% for grey matter water content).

Spectra were analysed using LCModel (Provencher 1993) that calculates the best fit to the experimental spectrum as a linear combination of model spectra (spectra of metabolite solutions). Seventeen metabolites were included in the basis set: alanine (Ala), aspartate (Asp), creatine (Cr), γ-aminobutyric acid (GABA), glucose (Glc), glutamate (Glu), glutamine (Gln), glycerophosphorylcholine (GPC), guanidoacetate (Gua), phosphorylcholine (PCho), myo-inositol (Ins), lactate (Lac), N-acetylaspartate (NAA), N-acetylaspartylglutamate (NAAG), phosphocreatine (PCr), scyllo-inositol, and taurine (Tau). Spectra of lipids and macromolecules were also included in the basis set. Only those metabolites that were estimated to have Cramer-Rao lower bounds (CRLB) less than 20%, which corresponded to an estimated concentration error <0.2 µmol/g, were included into the quantitative analysis. In some cases, metabolites that have resonance overlapped or very close are also given as their sum.

The signals due to inositol, glutamate and glutamine underwent J-coupling modulation with increasing TEs. However, the decreases in these signals due to J-coupling at TE = 23 ms were automatically accounted for in the LCModel basis sets.

### Statistical Analysis

To avoid litter effects, only one offspring per dam was used in each test. Additionally, in order to minimize potential carryover effects due to repeated testing, mice used for the behavioural observations during adolescence were tested either for the progressive ratio or for general locomotion and elevated-0-maze (E0M). This strategy of allocation of subjects to experimental groups was aimed at guaranteeing that at least seven unrelated individuals constituted the statistical unit. Thus, for the behavioural analysis we tested N = 7/8 subjects per prenatal treatment per drug; for the MRI/MRS, we tested N = 7 subjects per prenatal treatment per drug. The total number of subjects was n = 60. An independent group of subjects were tested for MRI/MRS. Data were analysed using general analysis of variance for split-plot designs. Post-hoc comparisons were performed using Tukey’s test. Statistical analysis was performed using Statview II (Abacus Concepts, CA, USA). Data are expressed as mean ± standard error of the mean (SEM). Significance level was set at p<0.05.

In particular, for the analysis of fluid intake the general model was 2 prenatal treatment (AFR vs. PNC) x 4 days. The general model for body weight gain was 2 prenatal treatment x 2 drug (VEH vs. URB597) x 9 time points. The general model for general locomotion was 2 prenatal treatment x 2 drug x 2 time points (P29 vs. P38). The general model for progressive ratio schedule was 2 prenatal treatment x 2 drug x 4 session days. Finally, the general model for the E0M, the open field and the different metabolites measured through MRI/MRS was 2 prenatal treatment x 2 drug. Prenatal treatment and drug were between-subject factors while all other variables were within-subject factors.

## Results

### Fluid Intake in Dams

To evaluate whether corticosterone supplementation resulted in variations in overall drinking, and to quantify the corticosterone ingested by each dam, we constantly monitored fluid intake in all dams during pregnancy. Levels of fluid intake were indistinguishable between dams having access to tap water and dams supplemented with corticosterone (12.54±0.55 ml and 13.20±0.66 ml respectively; prenatal treatment: F(1,19) = 0.07, NS). The daily average fluid intake shown by PNC dams resulted in 1.32±0.06 mg of corticosterone.

### Body Weight Gain in the Offspring

**Figure 3:**
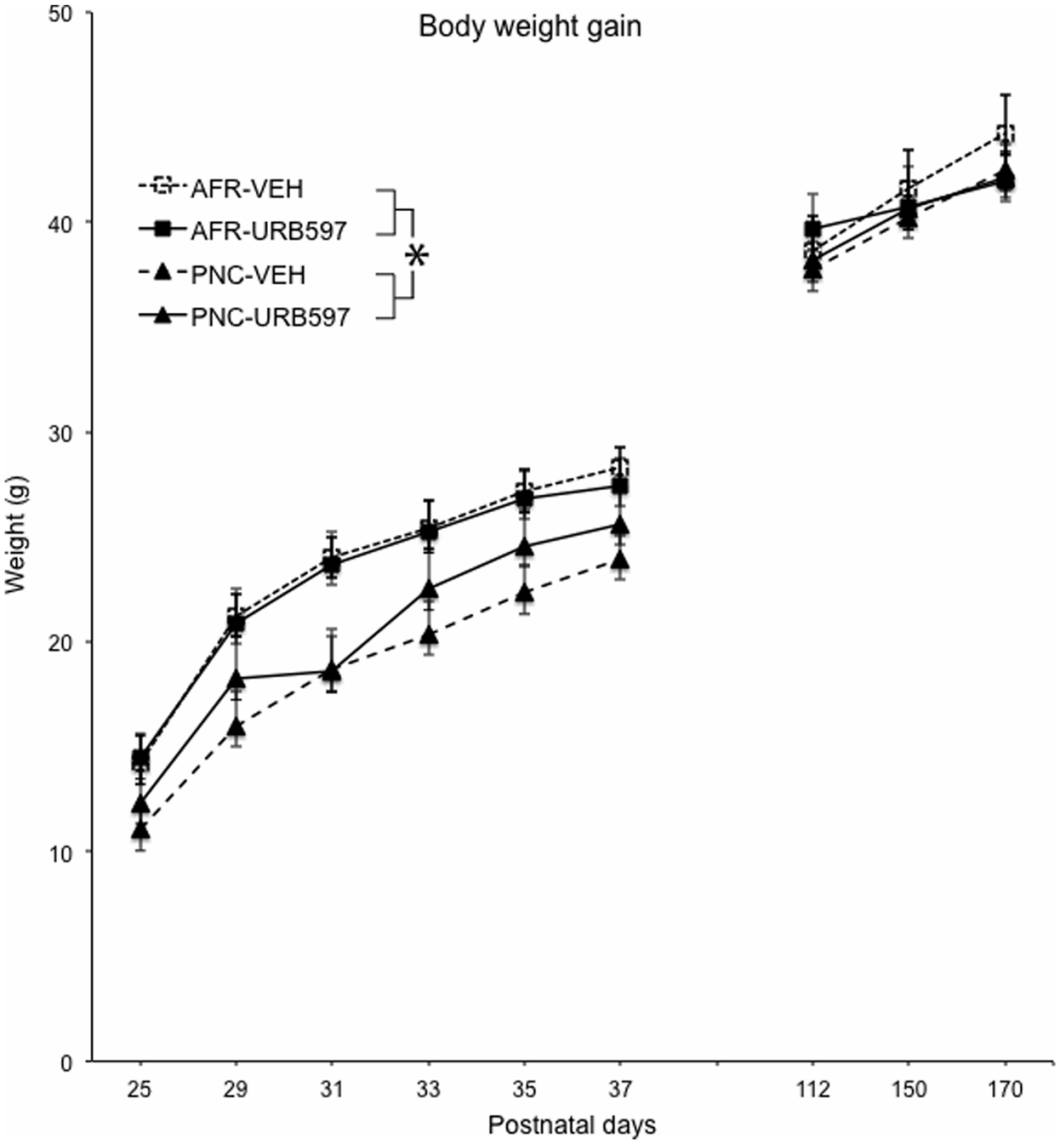
Body weight gain of mice exposed to corticosterone in the drinking water (100 mg/l) during the last week of gestation, and to a pharmacological modulation of the ECS during adolescence (URB597 i.p. administration - 0.4 mg/kg - between postnatal days 29–38). *p<0.05 significantly different from AFR subjects. (N = 7–8 per group) AFR = animal facility rearing; PNC = prenatal corticosterone administration; VEH = vehicle; URB597 = URB597 administration between P29–38).

Body weight was constantly monitored between infancy and adulthood in order to evaluate whether prenatal corticosterone and URB597 administration during adolescence directly affected offspring body growth. Prenatal corticosterone administration significantly reduced body weight between infancy through adolescence (see Fig. 3). Thus, developing mice prenatally exposed to corticosterone showed reduced body weight compared to AFR subjects (prenatal treatment: F(1,26) = 8.8, P = 0.018). The effects of prenatal corticosterone administration on body-weight were independent of the URB597 administration (prenatal treatment x drug: F(1,26) = 0.08, NS) and were limited to the early stages of development. Thus, adult PNC mice showed indistinguishable body weight compared to AFR controls (prenatal treatment in adulthood: F(1,26) = 1.3, NS). Additionally, URB597 administration had no effect on body weight gain. Thus, adolescent and adult mice that received URB597 administration for 10 consecutive days did not show significant variations in body weight compared to VEH-injected subjects (main effect of drug: F(1,26) = 0.04, NS).

### Short-term Effects of Acute and Semi-chronic URB597 Administration on General Locomotion

**Figure 4:**
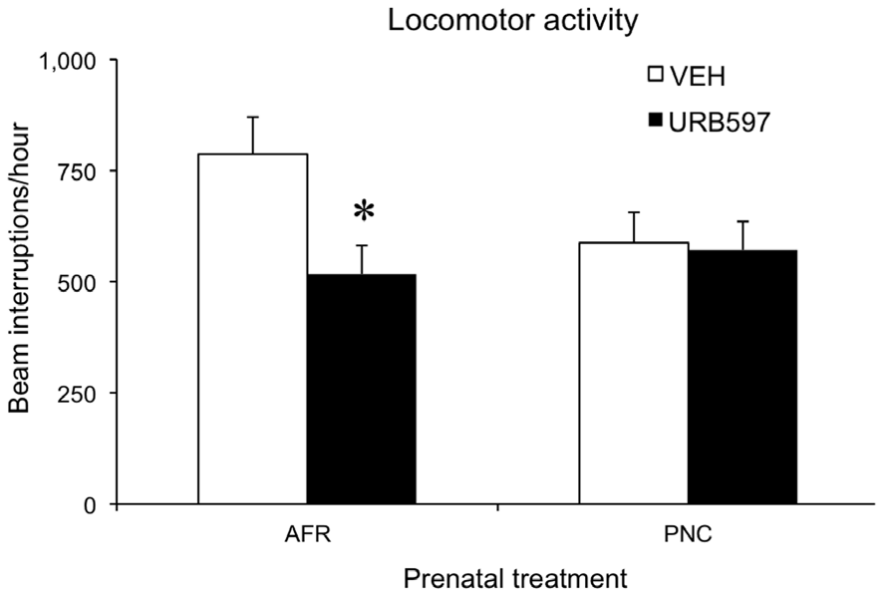
Spontaneous locomotion of adolescent mice (P29) measured in the home-cage during a single 5-hr session, starting 35 minutes after URB597 i.p injection. (N  = 8 per group). *p<0.05 significantly different from AFR-VEH in post-hoc tests. AFR = animal facility rearing; PNC = prenatal corticosterone administration; VEH = vehicle; URB597 = URB597 administration between P29–38).

Acute URB597 administration during adolescence significantly reduced absolute levels of general locomotion (main effect of drug: F(1,27) = 8.7, P = 0.006, see Fig. 4). Yet, the effects of URB597 administration were significant only in AFR and not in PNC mice (prenatal treatment x drug: F(1,27) = 8.5, P = 0.007). Thus, whilst AFR subjects showed reduced locomotion in response to an acute administration of URB597 on P29, PNC URB597-treated mice were indistinguishable from their VEH-injected controls (p<0.05 in post-hoc tests, see Fig. 4). The absence of differences within the PNC group may be due to a general moderate reduction in absolute levels of locomotion in PNC-VEH subjects. Absolute levels of general locomotion were also addressed following the last URB597 administration on P38. In response to the semi-chronic drug administration regimen, subjects from the four experimental groups, showed indistinguishable absolute levels of locomotion (prenatal treatment x drug F(1,27) = 0.4473, NS, data not shown).

### Long-term Consequences of Semi-chronic URB597 Administration on Anxiety-Related Behaviour, Locomotion, Anhedonia and Brain Metabolism

#### Elevated-0-maze (Fig. 5a)

**Figure 5:**
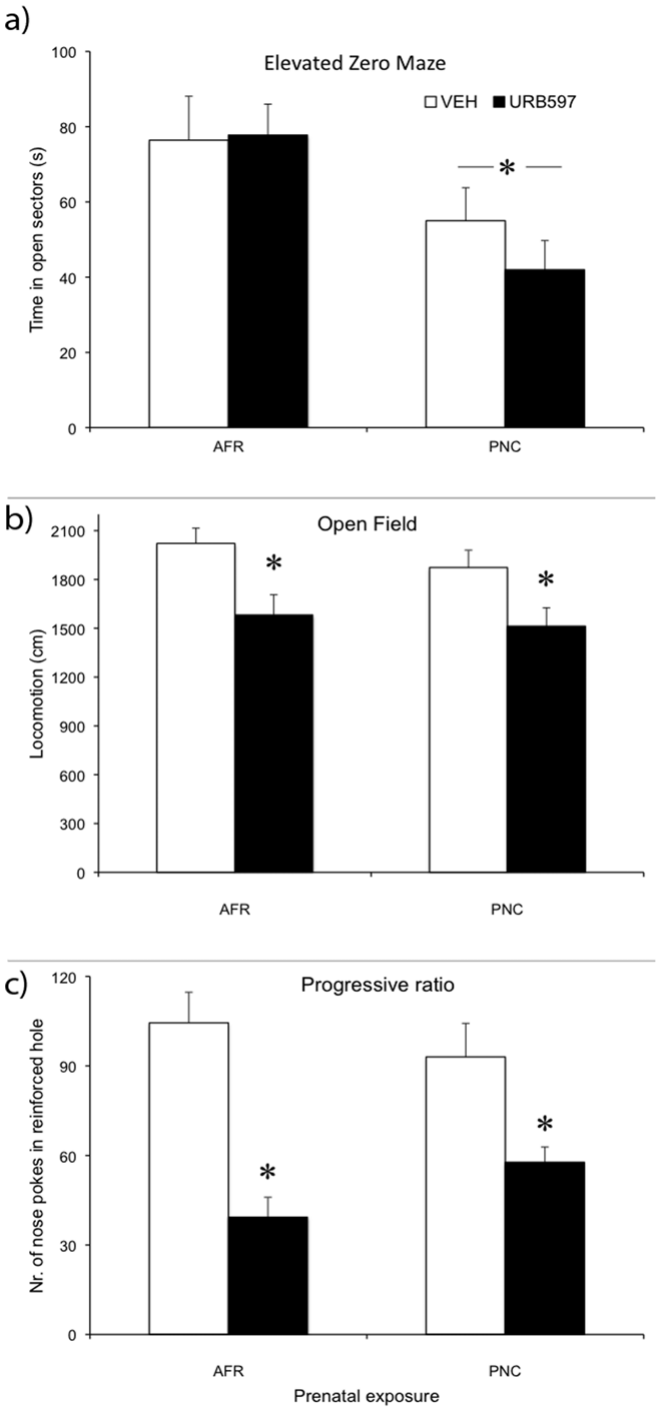
Behavioural responses of adult mice. (**a**) Time spent (s) in the open sectors of the 0-maze during a 5-min long test session. (N = 8 per group). *p<0.05 significantly different from AFR (significant main effect of prenatal treatment in the general ANOVA). (**b**) Distance traveled (cm) during the 30-min long test session of the open field. (N = 8 per group). *p<0.05 URB597 subjects significantly different from VEH subjects (significant main effect of drug in the general ANOVA). (**c**) Number of nose pokes in reinforced hole in the progressive ratio operant procedure. (N = 8 per group). *p<0.05 URB597 subjects significantly different from VEH subjects (significant main effect of drug in the general ANOVA).

Anxiety related behaviour was assessed in a single 5-min E0M experimental session. In the absence of significant differences due to URB597 administration during adolescence (drug: F(1,28) = 0.330, NS), adult mice exposed to corticosterone during gestation predictably showed a significant reduction in the time spent in the open sectors (prenatal treatment: F(1,28) = 7.306, p = 0.0115, see Fig. 5a) irrespective of the administration of URB597 during adolescence (prenatal treatment x drug F(1,28) = 0.491, NS). These results were complemented by increased time spent in the closed sectors of the E0M (main effect of prenatal treatment: F(1,28) = 7.306, p = 0.0115), irrespective of URB597 administration (drug: F(1,28) = 0.330, NS; prenatal treatment x drug F(1,28) = 0.491, NS).

Additionally, compared to AFR controls, PNC mice showed a reduced number of transitions between the open and the closed sectors of the E0M (main effect of prenatal treatment: F(1,27) = 8.591, p = 0.006). Finally, the administration of URB597 during adolescence also reduced the number of transitions between the open and the closed sectors of the E0M (main effect of drug: F(1,27) = 5.023, p = 0.033).

#### Open field activity (Fig. 5b)

Data on absolute levels of locomotion during the 30-min session of the open field revealed that the semi-chronic exposure to URB597 during adolescence had significant long-term effects. Specifically, in the absence of differences due to prenatal treatment (F(1,28) = 0.775, NS), adult mice exposed to URB597 during adolescence displayed reduced levels of locomotion compared to VEH-injected subjects (main effect of drug: F(1,28) = 4.226, p = 0.0492). Additionally, this effect was independent of prenatal treatment (prenatal treatment x drug F(1,28) = 0.073, NS). Finally, neither PNC nor URB597 administration resulted in variations in the time spent in the centre of the open field (prenatal treatment F(1,28) = 2.71, NS; drug F(1,28) = 0.883, NS; prenatal treatment x drug F(1,28) = 0.420, NS).

#### Progressive ratio (Fig. 5c)

In order to evaluate whether prenatal corticosterone exposure and pharmacological stimulation of the ECS during adolescence affected depressive-related behavioural responses, we assessed adult mice on a progressive ratio schedule. Thus, mice were required to nose poke to obtain a palatable reward in an operant task. Following three sessions of fixed FR1 and three sessions of FR3, mice were required to perform an increasing number of operant responses to obtain the reward. During the first sessions of the training phase (FR1), URB597 mice showed a reduced number of nose-pokes compared to VEH individuals (drug: F(1, 21) = 4.554, p = 0.045). Yet, such difference disappeared during the FR3 phase (drug: F(1, 21) = 3.75, NS), thus indicating that all mice achieved a similar level of operant responding before the beginning of the PR phase. The break point achieved, the number of operant responses and the number of rewards obtained were significantly reduced in URB597 mice (drug: F(1,21) = 4.81, p = 0.040; F(1,21) = 4.85, p = 0.039; F(1,21) = 4.80, p = 0.040, respectively). Prenatal corticosterone administration did not influence these differences (prenatal treatment x drug: F(1,21) = 0.603, p = 0.446 (breakpoint); F(1,21) = 0.432, p = 0.639 (number of operant responses); F(1,21) = 1.716, p = 0.204 (number of rewards)).

### Magnetic Resonance Imaging-guided Spectroscopy in Selected Brain Areas

The quantitative results of these analyses are summarised in Table 1 and in Table S1. Water T2 analyses confirmed that no changes occurred in the T2s within the groups in prefrontal cortex, hippocampus and hypothalamus (prenatal treatment x drug: F(1,23) = 0.094, p = 0.76; F(1,22) = 2.094, p = 0.16; F(1,22) = 0.899, p = 0.35 respectively, data not shown).

**Table 1:** Levels of metabolites in selected brain areas.

	Prefrontal cortex				Hypothalamus				Hippocampus			
	AFR		PNC		AFR		PNC		AFR		PNC	
	VEH	URB597	VEH	URB597	VEH	URB597	VEH	URB597	VEH	URB597	VEH	URB597
*NAA*	5.39±0.2	4.75±0.4	7.21±1	4.90±0.1	5.71±0.3	5.53±0.3	4.57±0.4*	5.43±0.2	7.41±0.2	7.56±0.2	7.29±0.4	7.28±0.3
Cr+PCr	6.46±0.4	5.68±0.3	5.71±1.6	5.70±0.2	6.61±0.4	6.3±0.2	5.92±0.2	6.67±0.3	8.84±0.1	8.90±0.2	8.99±0.2	9.15±0.3
Gln	5.45±0.6	5.98±0.8	7.06±0.8	4.71±0.9	5.66±0.5	5.93±0.4	5.74±0.6	5.19±0.6	5.26±0.2	4.62±0.3	5.38±0.4	4.78±0.4
Tau	10.5±0.5	8.31±0.3*	10.3±0.8	7.98±0.6	4.05±0.4	3.89±0.4	3.72±0.6	3.34±0.7	11.4±0.5	10.7±0.7	9.56±0.6*	9.82±0.5
Glu	8.92±0.8	6.67±0.7	11.4±1.2	7.64±0.3	7.49±0.4	7.07±0.5	8.08±0.5	7.03±0.4	8.14±0.2	8.37±0.2	8.92±0.5*	9.03±0.1
Ins	5.41±0.4	4.30±0.4*	4.79±0.9	3.71±0.3	6.96±0.2	7.16±0.3	6.04±0.2*	6.25±0.3	6.11±0.4	6.08±0.4	5.42±0.4	5.74±0.4
GPC+PCho	1.14±0.1	1.16±0.2	1.38±0.2	1.13±0.1	2.18±0.1	2.17±0.1	2.27±0.1	2.44±0.1	1.42±0.7	1.5±0.04	1.5±0.07	1.6±0.06
NAA+NAAG	5.76±0.4	5.11±0.4	7.40±0.6	4.95±0.1	6.48±0.2	6.22±0.3	5.95±0.3	6.53±0.2	7.82±0.2	7.94±0.2	8.20±0.3	7.63±0.3
Glu+Gln	13.2±1.6	11.5±1.2	10.2±0.8	15.6±0.7	12.8±0.6	13.0±0.6	12.2±0.5	12.7±0.3	13.6±0.3	12.9±0.5	13.9±0.3	13.7±0.8

Levels of metabolites (mM units) in prefrontal cortex (PFC), hypothalamus and hippocampus of adult mice, measured through MRI/MRS. *p<0.05 significantly different from AFR-VEH in post-hoc tests. AFR = animal facility rearing; PNC = prenatal corticosterone administration; VEH = vehicle; URB597 = URB597 administration between P29–38).

#### Prefrontal cortex (Fig. 6a,b)

Whilst prenatal corticosterone exposure had no effect on metabolites’ concentration in the prefrontal cortex, URB597 administration during adolescence resulted in significant long-term reductions in inositol (drug: F(1,18) = 11.97, p = 0.0023; prenatal treatment x drug F(1,18) = 0.001, NS) and taurine (drug: F(1,18) = 15.070, p = 0.001; prenatal treatment x drug F(1,18) = 0.007, NS) concentrations.

**Figure 6:**
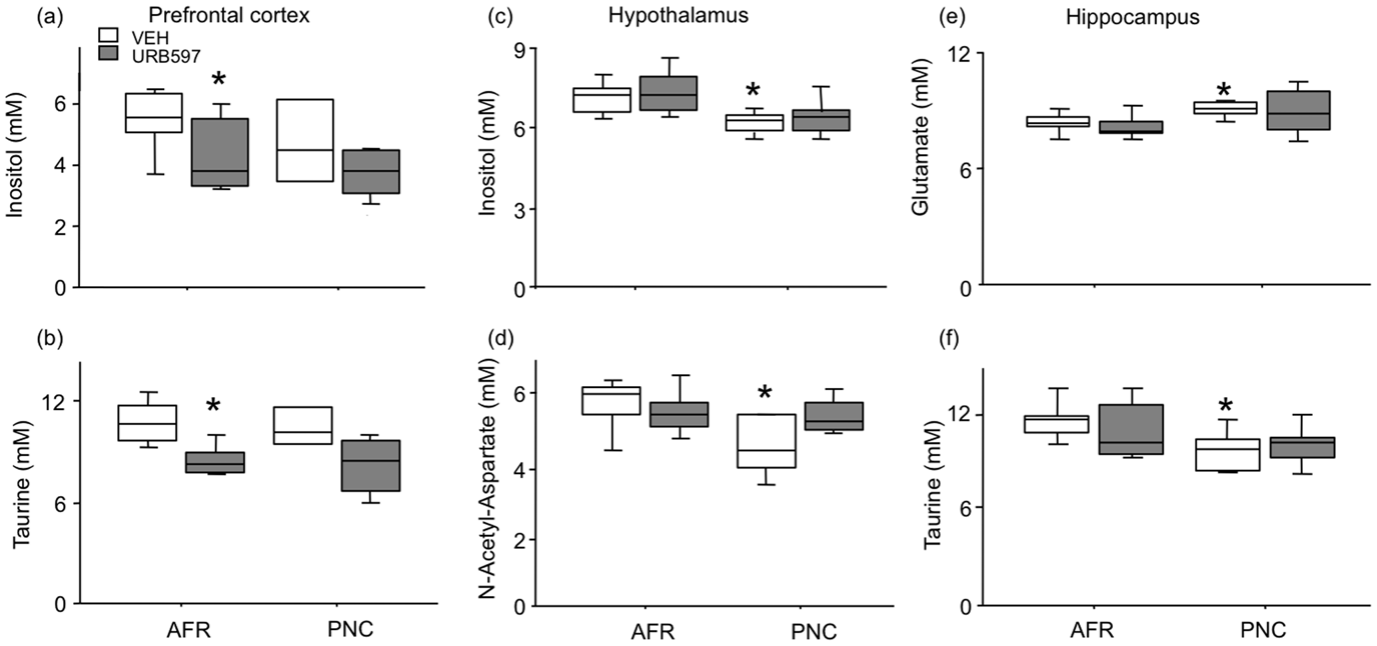
Levels of metabolites (mM units) – expressed as median, and upper and lower quartiles (N = 7 per group) – in prefrontal cortex (inositol, a, and taurine, b), hypothalamus (inositol, c, and NAA, d) and hippocampus (glutamate, e, and taurine, f) of adult mice, measured through MRI/MRS. *p<0.05 significantly different from AFR-VEH in post-hoc tests. AFR = animal facility rearing; PNC = prenatal corticosterone administration; VEH = vehicle; URB597 = URB597 administration between P29–38).

#### Hypothalamus (Fig. 6c,d)

In the absence of differences related to URB597 administration during adolescence, we observed that PNC subjects showed reduced concentrations of inositol (drug: F(1,23) = 4.185, p = 0.016; prenatal treatment x drug F(1,23) = 1.66, NS) and NAA compared to AFR controls (drug: F(1,22) = 5.041, p = 0.035; prenatal treatment x drug F(1,22) = 3.51, P = 0.074). The other metabolites were apparently unaffected by prenatal corticosterone administration.

#### Hippocampus (Fig. 6e,f)

In line with what we observed in the hypothalamus, URB597 administration during adolescence did not modify the concentrations of all the metabolites investigated in the hippocampus. Conversely, prenatal corticosterone exposure permanently modified relative concentrations of Glu and taurine in the hippocampus. Specifically, compared to AFR individuals, PNC adult mice showed increased concentrations of Glu (drug: F(1,23) = 4.64, p = 0.04; prenatal treatment x drug F(1,23) = 0.021, NS) and reduced concentrations of taurine (drug: F(1,23) = 5.992 p = 0.022; prenatal treatment x drug F(1,23) = 0.73, NS).

## Discussion

In the present study, we showed that both prenatal exposure to corticosterone (PNC) and cannabinoid URB597 administration during adolescence significantly modify emotional regulations during development and alter, in the long-term, several indices of brain metabolism. Specifically, PNC individuals showed substantial reductions in body weight during infancy, increases in behavioural indices of anxiety in the E0M, and alterations in inositol and NAA concentrations in the hypothalamus, and taurine in the hippocampus (coupled with increased levels of Glu). URB597 subjects showed significant reductions in general locomotion, operant responding to obtain palatable rewards and in inositol and taurine concentrations in the prefrontal cortex. Thus, both treatments significantly affected long-term regulations in the experimental individuals; yet, whilst we originally hypothesised these effects to be synergistic, they were independent.

The consequences of prenatal stress have been addressed, in literature, using a number of different paradigms, ranging from psychological and physical stressors (repeated exposure to restraint stress [40], [41], [48], [49] and exposure to novelty [50]), to hormonal supplementation (exogenous administration of glucocorticoids [37], [51], [52]). Both paradigms have advantages and disadvantages. The former have the advantage of adopting psychological stressors, likely to elicit an integral sympathetic- and HPA-mediated stress response, thus mimicking the effects of a stressful pregnancy; yet, their main disadvantage is that repeated exposures to a given stressor may result in a habituation profile (see e.g. [30]) shown by the individuals and by the fact that each subject can display differential responses to the stressor. Therefore, the amount of stress perceived can vary both between individuals and across days. Complementarily, although the use of hormonal supplementation only mimics a single aspect of the integral stress response, it can be controlled and tailored to each given experimental group.

The possibility that the effects of prenatal stress on emotional regulation depend on stress-induced elevations in circulating levels of corticosterone has been cogently described by Weinstock [7]. In line with this hypothesis, Salomon and colleagues first showed that maternal adrenalectomy during gestation abolished most of the effects of prenatal stress; and then that some of these effects were reinstated by corticosterone administration [37]. Based on these findings, on its easier manageability, and on our previous experience with these paradigms [36], [38], we favoured the use of exogenous corticosterone supplementation in the maternal drinking water.

### Short and Long-term Consequences of Prenatal Corticosterone Exposure

In line with previous literature findings [53]–[55], exogenous corticosteroid administration during the late stages of gestation resulted in a significant reduction in body weight. Present data are in agreement with O’Regan and colleagues [54] that reported that dexamethasone administration during the last third of pregnancy resulted in a transient reduction in offspring body weight. The difference between dexamethasone-treated and control subjects was absent in adulthood [54]. Additionally, Waddell and colleagues observed that prenatal dexamethasone administration resulted in a significant increase in corticosterone secretion in adult rats [53]. Similar results were also obtained by Hauser and colleagues [52]. Gao and colleagues [56] proposed that the effects of prenatal stress on offspring’s body weight may relate to maternal malnutrition and to significant reductions in serum levels of insulin-like growth factor-1, growth hormone and prolactin. Additionally, the long-term reductions in body weight may also be indirectly mediated via PNC-dependent modulation of HPA reactivity. Specifically, several studies [52], [53], [57] demonstrated that prenatal corticosteroid administration persistently modifies HPA activity both in basal conditions and in response to psychological stress. Since the HPA axis directly regulates metabolic and anabolic processes, we offer that the transient effects of prenatal stress on body weight may also relate to its indirect effects on corticosteroid activity [58].

The possibility that prenatal corticosterone administration persistently adjusted HPA-mediated phenotypes is in accordance with the results obtained in the E0M. Thus, prenatal exposure to corticosterone was associated with a significant increase in anxiety-related behaviour in the adult offspring as shown by the reduced time spent in the open sectors of the E0M. Given the complex experimental design of this study, we were not able to directly measure corticosterone reactivity in adult naïve subjects. Despite the presence of previous observations [52] supporting the possibility that prenatal glucocorticoids may increase corticosterone reactivity in adulthood, future studies are needed to clarify this hypothesis. “Anxiogenic” profiles in adult offspring exposed to prenatal stress have been long known [8], [37], [40], [49], [57], [59]. Vallée and colleagues [49] observed that the effects of prenatal stress on adult emotionality correlate with variations in corticosterone reactivity, whereby the time spent in the open arms of the elevated plus maze inversely correlated with plasma corticosterone concentrations in response to restraint stress. In line with this view, Hauser and collaborators [52] observed that HPA reactivity was elevated in adult offspring born to dams exposed to dexamethasone during the last third of pregnancy. We note, however, that the data observed in the E0M were not paralleled by the time spent in the centre of the open field, which did not vary among the different experimental groups. Despite the fact that the time spent in the centre of the open field constitutes a valid indicator of anxiety [60], several studies reported that it can be unrelated to the time spent in the open sectors of the E0M. Specifically, factor analyses demonstrated that the anxiety-related indicators of the different tests loaded on independent factors [61]–[63]. These analyses therefore suggested the anxiety-related variables measured in these tests have a substantial degree of independence and may even reflect different behavioural dimensions [62]. Likewise, PNC mice showed reduced indices of locomotion on the E0M (number of transitions between the closed and open arms) but not in the open field. Although the number of transitions in the elevated plus maze (EPM) constitute a valid indicator of general locomotion [64], the number of transitions in the E0M may not reflect an analogous dimension. Specifically, the nature of the E0M is fundamentally different from the EPM whereby it lacks the central platform. The latter physically allows rodents to move between closed arms without entering the open arms (i.e. they can move from one closed arm to the central platform and than back to the same or forward to the opposite closed arm). Such possibility is precluded in the E0M, which does not feature an intermediate zone. Therefore, transitions in the E0M always require mice to explore the open arms. This fundamental difference may explain why locomotion in the open field does not correlate with the number of transitions observed in the E0M. Future multivariate analyses [64] are needed to clarify this aspect.

In partial contrast with our expectations, prenatal corticosterone exposure did not result in significant alterations in progressive ratio responding. In the light of the putative role of prenatal stress in inducing depressive-like symptoms (e.g. [10], [57]) we originally predicted early exposure to an elevated dosage of corticosterone to reduce motivation towards palatable rewards. Yet, adult PNC mice failed to show increased indices of anhedonia. Literature data with respect to the relationship between prenatal stress and depressive-like behaviours are conflicting. Specifically, whereas some authors observed a significant increase in depressive like behaviours in response to prenatal stress (e.g. [65] [8]), some others failed to replicate these findings [66], [67]. Our results are analogous to those obtained by Hauser and colleagues [52] that did not observe variations in progressive ratio responding in adult rats exposed to dexamethasone during gestation.

In order to map behavioural differences onto brain metabolic alterations, we addressed MRI/MRS in adult offspring. We observed that NAA levels were significantly reduced in the hypothalamus of PNC mice. NAA has been proposed to constitute a marker of neuronal number and viability [68] and its levels have been shown to fluctuate in response to prenatal stress [69]. Furthermore, a recent finding demonstrates that elevated levels of corticosterone concentrations in rats are associated with reductions in NAA levels in stress-sensitive brain areas [70]. Here we observed that NAA concentrations are reduced in a brain area directly involved in emotional processing. This result is in agreement with several studies conducted in different species (ranging from Tree shrews [71] to humans [72]). Thus, although hypothetical, our data support the view that PNC relates to an altered functionality of the HPA axis and may thus partly explain the aforementioned increase in anxiety-related behaviour shown by PNC mice. As a further corroboration of this hypothesis, we also observed that inositol (a glial marker [73]) was reduced in PNC adult mice.

PNC mice also showed significantly increased glutamate and significantly decreased taurine in the hippocampus. With respect to the former, its widely distributed action as a neurotransmitter in the brain confers on it a fundamental role in neuronal excitability. An abnormal increase in glutamate has been associated with excitotoxicity [74] and, in turn, with the onset of neuropsychiatric disorders [75]. Specifically, Gorman and Docherty [76] proposed that stressful life events may first increase excitotoxic glutamatergic transmission and then affect dendritic spine density. These sequelae of events have been associated with mood and anxiety related disorders [76]. Conversely, taurine has been proposed to exert neuroprotective actions in neural tissue [77], and act as an anti-anxiety agent in the central nervous system [78]. Present results are in full agreement with a recent study performed by Barbosa Neto and colleagues [79]. Specifically, the authors investigated *post-mortem* hippocampal metabolic profiles in adult rats exposed to early stress (24-hr maternal deprivation on P11) and observed significant increments in glutamate and significant reductions in taurine concentrations [79]. Thus, the metabolic profile observed in the hypothalamus and in the hippocampus of PNC mice constitutes a potential mediator of the increased anxiogenic profile observed in the adult offspring.

Although these effects are most likely mediated by the direct elevation in corticosterone concentrations in the dams [37], it cannot be excluded that they were also partly mediated by gestational stress-dependent alterations in maternal care [80]. Hauser and colleagues addressed this possibility by evaluating emotional and cognitive abilities in adult rats exposed to dexamethasone during gestation, and cross-fostered to untreated dams at birth [51], [52]. Whilst some of the effects were solely dependent on prenatal dexamethasone, some others were explained by the combination of prenatal and postnatal factors. Further studies, involving cross-fostering procedures are needed to clarify this aspect [81]–[83].

### Short- and Long-term Consequences of Pharmacological ECS Stimulation during Adolescence

One of the main goals of this study was to demonstrate that exposure to cannabinoid agonists during adolescence may relate to alterations in emotional reactivity in adulthood. This hypothesis has been recently reviewed by Casadio and collaborators [84], who described a Swedish cohort study reporting an association between early cannabis use and later onset of schizophrenia in adulthood [85]. Additionally, Patton and colleagues (2002) reported that heavy cannabis use in adolescence related to a fivefold increased risk of reporting a state of depression and anxiety early in adulthood [86].

To investigate the consequences of ECS modulation during adolescence, rather than using a direct cannabinoid agonist, we used an indirect agonist acting through the inhibition of the fatty acid amide hydrolase, the enzyme catalysing the degradation of anandamide. Compared to direct agonists, the administration of URB597 allowed modulating the ECS within a physiological range. Thus, rather than directly binding CB receptors, URB597 inhibits the degradation of endocannabinoid ligands whenever they are naturally elevated on demand, ultimately protracting their action [39]. Additionally, experimental evidence demonstrates that URB597 has direct effects on emotional regulations [87]–[88]. Finally, we used this compound in order to compare present evidence with previous studies in which we administered URB597 to adolescent rodents [39].

In line with our expectations, URB597 administration resulted in a short-term reduction of general locomotion. Beside the well-known effects of direct cannabinoid agonists on locomotor activity in rodents [89] and humans [90], Lee and colleagues showed that URB597 [91] was sufficient to reduce locomotor activity in rats. In line with present findings, Eisenstein and collaborators [92] observed that the effects of URB597 on general locomotion, attributed to CB1-dependent mechanisms, dissipated over time. Specifically, adopting a semi-chronic exposure to URB597 (eight days), they observed that whereas a single exposure to URB597 resulted in reduced locomotion, repeated administrations were ineffective. Although the semi-chronic administration of URB597 had no significant effect on general locomotion in the short-term (i.e. towards the end of adolescence), it resulted in a significantly reduced locomotor activity in the long-term. The latter has often been associated with depressive-related profiles [93]. This observation is further corroborated by the significantly increased anhedonia shown by URB597 adult mice. Specifically, along with the effects of URB597 administration on general locomotion, we also observed that adolescent exposure to URB597 reduced the number of operant responses to obtain a reward later in adulthood. This finding is in agreement with a previous study demonstrating that chronic exposure to a cannabinoid agonist during adolescence was sufficient to increase anhedonia in adult rats [94]. The same study also revealed that a similar cannabinoid administration during adulthood had no effect on progressive ratio responding. Likewise, an independent study also demonstrated that a chronic administration of a cannabinoid receptor agonist (WIN55,212) during adolescence resulted in a depressive-like phenotype in the forced swim test [95]. These alterations have been associated with potential persistent variations at the level of several neurotransmitter systems, including the opioid [96], the endocannabinoid and the dopaminergic [94]. In line with this hypothesis, we recently observed that a URB597 regime analogous to that used in the present study, persistently downregulated CB1 receptors in reward-related dopaminergic brain areas (i.e. nucleus accumbens, ventral tegmental area and hippocampus) [97].

The role of cannabinoid receptors in the acquisition and preservation of operant responding for palatable rewards has been detailed by Ward and Dykstra (2005). Specifically, they observed that, compared to wild-type controls, CB1 receptor knockout mice required a longer number of sessions to acquire operant responding and that the break point achieved was significantly reduced [98]. Additionally, the long-term effects of URB597 administration on progressive ratio responding may be related to treatment-dependent alterations at the level of the prefrontal cortex. Here we observed that adult mice exposed to URB597 during adolescence showed significantly reduced levels of taurine and inositol in the prefrontal cortex. Inositol has been proposed to constitute an osmoregulator in primary astrocytes and to contribute to the maintenance of brain volume [99]. Its reduction has been linked to glial loss or altered glial metabolism [100]. Additionally, reductions in myo-inositol levels have been observed in depressed patients, thus suggesting a role for this metabolite in the clinical course of depression [101]. Thus, the reduction in inositol concentrations, coupled with the altered neuroprotection associated with the reduction in taurine [77], [78] may indirectly support the view that the prefrontal cortex was dysfunctional in adult mice treated with URB597 during adolescence. In line with this hypothesis, Moscarello and colleagues [102] observed that the temporary inactivation of the prefrontal cortex resulted in reduced break points in rats tested on a progressive ratio schedule analogous to that used in the present study. We recently performed a similar study in adult rats exposed to prenatal stress – in the form of repeated restraint stress administered to the dams – and to URB597 administration during adolescence [39]. In contrast with present observations, we only observed variations in the glutamatergic metabolic profile in the hippocampus. Although we cannot unequivocally explain the conflicting findings, we believe that methodological aspects (i.e. animal species and different forms of prenatal stress) may partly account for them.

Finally, present data do not support the view that cannabinoid exposure during adolescence may result in long-term increase in anxiety-related behaviour (e.g. [103]). Specifically, we failed to observe alterations in the time spent in the open arms of the E0M in adult mice treated with URB597 during adolescence. This result is in line with previous studies addressing the long-term effects of cannabinoid agonist administration during adolescence on anxiety-related behaviours [94], [95]. Thus, in both these studies adolescent rats were exposed to WIN 55,212 and then analysed for anxiety-related behaviours in an open field [94] or in a novelty-induced suppression of food intake test [95]. None of these studies reported long-term differences as a function of previous exposure to the cannabinoid agonist. The absence of significant differences between treated and control rats in independent studies that adopted different test procedures supports the view that cannabinoid administration in adolescent rodents does not induce major long-term alterations in anxiety-related responses.

### Concluding Remarks

In contrast with our predictions, rather than being interactive in nature, the effects of the developmental interventions were independent from each other. Specifically, based on the cross-talk between the ECS and the HPA axis [27], [28] in the regulation of emotions, we hypothesised that the effects of URB597 administration during adolescence would exacerbate the consequences of prenatal stress. Yet, these effects were independent. Although we cannot unequivocally explain this result, we offer several non-mutually exclusive hypotheses. First, in some instances, floor effects may explain the absence of additive consequences of PNC and URB597: specifically, with respect to general locomotion and to the time spent in the open sectors of the E0M, it is plausible that PNC subjects achieved values of a magnitude sufficiently small not to be further exceeded by additional treatments. Similar reasoning may also pertain to some of the effects we observed on brain metabolism. Specifically, we hypothesise that, together with direct effects on the HPA axis, prenatal corticosterone administration may impact the ECS and, in turn, reduce the likelihood to observe further modifications associated with additional elevations of endocannabinoid transmission. In line with this hypothesis, Fride and collaborators [104] recently proposed that prenatal stress may directly affect the ECS. Second, it is possible that PNC and URB597 differently regulated the individual phenotype through independent mechanism and that such fundamental distinction in the biological determinants results in the observed findings. Third, we cannot exclude the possibility that the use of a direct and more potent cannabinoid agonist and/or higher doses of prenatal corticosterone may trigger stronger consequences. Further studies are needed to clarify these points.

## Supporting Information

Table S1 Levels of metabolites (median (interquartile range)) in prefrontal cortex (PFC), hypothalamus and hippocampus of adult mice, measured through MRI/MRS. *p<0.05 significantly different from AFR-VEH in post-hoc tests. AFR = animal facility rearing; PNC = prenatal corticosterone administration; VEH = vehicle; URB597 = URB597 administration between P29–38.
(DOC)
