# Supplementary material for: Prenatal Stress and Peripubertal Stimulation of the Endocannabinoid System Differentially Regulate Emotional Responses and Brain Metabolism in Mice
Source: PLoS One. 2012 Jul 25;7(7):e41821. doi: 10.1371/journal.pone.0041821 (PMC3405010; doi:10.1371/journal.pone.0041821)
Supplement: Table S1 — Levels of metabolites (median (interquartile range)) in prefrontal cortex (PFC), hypothalamus and hippocampus of adult mice, measured through MRI/MRS. *p<0.05 significantly different from AFR-VEH in post-hoc tests. AFR = animal facility rearing; PNC = prenatal corticosterone administration; VEH = vehicle; URB597 = URB597 administration between P29–38. (DOC) [file pone.0041821.s001.doc]

**Table S1: Levels of metabolites in selected brain areas**

|  |  | Prefrontal cortex | | | |  | Hypothalamus | | | |  | Hippocampus | | | |  |  |
| --- | --- | --- | --- | --- | --- | --- | --- | --- | --- | --- | --- | --- | --- | --- | --- | --- | --- |
|  |  | AFR | | PNC | |  | AFR | | PNC | |  | AFR | | PNC | |  |  |
|  |  | VEH | URB597 | VEH | URB597 |  | VEH | URB597 | VEH | URB597 |  | VEH | URB597 | VEH | URB597 |  | |
| NAA |  | 5.62 (5.05-5.84) | 4.84  (4.57-5.68) | 6.80  (5.87-8.66) | 4.94 (4.77-5.25) |  | 6.03 (5.43-6.22) | 5.44 (5.09-5.77) | 4.40* (3.94-5.43) | 5.24 (5.01-5.79) |  | 7.28 (7.00-7.91) | 7.50 (7.35-7.78) | 7.35 (7.18-7.63) | 7.34 (6.58-7.94) |  | |
| Cr+PCr |  | 6.50 (5.95-7.02) | 5.45  (4.68-6.75) | 4.74  (3.36-6.93) | 5.71  (5.29-5.94) |  | 6.49 (5.99-7.46) | 6.26 (5.93-6.63) | 5.86 (5.68-6.35) | 6.56 (6.21-7.38) |  | 8.82 (8.62-9.17) | 8.93 (8.63-9.33) | 8.54 (8.10-8.97) | 8.88 (8.67-9.79) |  | |
| Gln |  | 5.40  (4.56-6.34) | 6.50  (4.92-7.05) | 7.06  (6.58-7.28) | 4.71  (3.79-5.64) |  | 6.10 (4.32-6.63) | 5.78 (5.12-6.58) | 5.74 (5.13-6.35) | 5.11 (3.91-6.46) |  | 5.09 (4.75-5.72) | 4.43 (4.07-5.23) | 5.01 (4.94-5.92) | 4.94 (4.39-5.67) |  | |
| Tau |  | 10.4 (9.47-11.4) | 8.08* (7.61-8.71) | 9.92 (9.26-11.36) | 8.23 (6.55-9.42) |  | 4.18 (3.63-4.81) | 3.59 (3.26-4.60) | 3.72 (3.16-3.83) | 3.12 (2.50-4.25) |  | 11.4 (10.5-11.7) | 9.96 (9.23-12.35) | 9.58* (8.22-10.2) | 9.94 (9.07-10.3) |  | |
| Glu |  | 8.85 (7.76-10.9) | 5.66 (5.26-8.05) | 10.68 (8.75-13.27) | 7.58 (7.27-7.68) |  | 7.28 (6.77-8.35) | 6.73 (6.30-7.40) | 8.10 (6.96-8.95) | 7.26 (6.65-7.70) |  | 8.36 (8.15-8.70) | 7.98 (7.81-9.07) | 9.09* (8.80-9.38) | 8.87 (8.02-10.0) |  | |
| Ins |  | 5.55 (5.04-6.29) | 3.77*  (3.32-5.49) | 4.47  (3.45-6.13) | 3.78 (3.06-4.46) |  | 7.05 (6.41-7.28) | 7.06 (6.52-7.73) | 6.11* (5.77-6.31) | 6.25 (5.73-6.45) |  | 6.12 (5.25-6.92) | 6.10 (5.28-6.44) | 5.67 (4.52-6.20) | 5.17 (5.00-5.17) |  | |
| GPC+PCho |  | 1.21 (0.89-1.29) | 1.03  (0.95-1.15) | 1.38  (1.25-2.08) | 1.19 (1.01-1.28) |  | 2.20 (1.89-2.39) | 2.12 (1.95-2.41) | 2.23 (2.14-2.31) | 2.33 (2.25-2.61) |  | 1.41 (1.29-1.50) | 1.50 (1.45-1.63) | 1.49 (1.44-1.62) | 1.52 (1.48-1.72) |  | |
| NAA+NAAG |  | 5.88 (5.12-6.63) | 5.25  (4.75-6.02) | 7.68  (6.09-8.88) | 4.94  (4.77-5.25) |  | 6.61 (6.32-6.89) | 6.27 (5.72-6.65) | 6.21 (5.17-6.59) | 6.53 (6.03-7.02) |  | 7.92 (7.48-8.19) | 7.85 (7.75-8.19) | 8.09 (7.72-8.92) | 7.96 (7.00-8.09) |  | |
| Glu+Gln |  | 14.2 (5.82-15.0) | 11.7 (9.05-12.34) | 14.9 (14.6-16.7) | 10.1 (8.75-11.0) |  | 12.9 (11.6-13.8) | 12.59 (11.2-14.1) | 12.5 (12.5-13.3) | 11.9 (11.4-11.9) |  | 13.8 (12.9-14.2) | 12.7 (11.9-14.1) | 14.1 (13.3-14.4) | 13.8 (12.6-15.3) |  | |
